# Carnosine induces intestinal cells to secrete exosomes that activate neuronal cells

**DOI:** 10.1371/journal.pone.0217394

**Published:** 2019-05-28

**Authors:** Yuka Sugihara, Shiori Onoue, Kosuke Tashiro, Mikako Sato, Takanori Hasegawa, Yoshinori Katakura

**Affiliations:** 1 Graduate School of Bioresources and Bioenvironmental Sciences, Kyushu University, Nishi-ku, Fukuoka, Japan; 2 Graduate School of Systems Life Sciences, Kyushu University, Nishi-ku, Fukuoka, Japan; 3 Faculty of Agriculture, Kyushu University, Nishi-ku, Fukuoka, Japan; 4 R&D Center, NH Foods, Ltd., Tsukuba, Ibaraki, Japan; Niigata Daigaku, JAPAN

## Abstract

Recently, we showed that imidazole dipeptide such as carnosine contained abundantly in chicken breast meat improves brain function in a double-blind randomized controlled trial. However, the underlying molecular mechanisms remain unknown. Here, we investigated whether carnosine activates intestinal epithelial cells and induces the secretion of factors that activate brain function. We focused on exosomes derived from intestinal epithelial cells as mediators of brain-gut interaction. Results showed that exosomes derived from Caco-2 cells treated with carnosine significantly induced neurite growth in SH-SY5Y cells. To clarify the molecular basis of this finding, we performed integrated analysis of microRNAs (miRNAs) with altered expression in exosomes in response to carnosine treatment and mRNAs with altered expression in target cells in response to exosome treatment to identify related miRNAs and their target genes. The combination of miR-6769-5p and its target gene *ATXN1* was found to be involved in the exosome-induced activation of neuronal cells.

## Introduction

Carnosine, an imidazole dipeptide, has been reported to exert anti-fatigue, anti-oxidant, and exercise performance-enhancing effects [1,2]. Recently, we reported that carnosine and its related peptide anserine improved brain function, as evidenced by experiments using a mouse model of Alzheimer’s disease [3] and a double-blind randomized controlled trial [4]. The expression of inflammatory cytokine CCL24 was suppressed in peripheral blood mononuclear cells from elderly individuals who had ingested carnosine/anserine, suggesting that the latter is a key molecule responsible for the observed effect [5]. Notably, we further demonstrate that anserine/carnosine supplementation preserves brain structure and function in elderly individuals carrying *APOE4* [6].

Although carnosine significantly improves brain function, the underlying molecular mechanisms remain unknown. As carnosine is degraded into β-alanine and l-histidine by carnosinase in serum [7], carnosine itself is not thought to be directly transported to the brain. Accordingly, we tested the alternative possibility that carnosine activates intestinal epithelial cells and induces the secretion of factors that penetrate the blood-brain barrier and activate neuronal cells. We have previously shown that carnosine activates intestinal epithelial cells [8], and suggested that this carnosine-induced activation of intestinal epithelial cells results in brain-gut interaction via the secretion of brain-derived neurotrophic factor [9]. In the present study, we targeted exosomes derived from intestinal epithelial cells as mediators that activate the brain-gut interaction. Further, we sought to clarify the involvement of exosomes in the carnosine-induced activation of the brain-gut interaction.

## Materials and methods

### Cell culture and reagents

We used a human colon cancer cell line, Caco-2, and a human neuroblastoma cell line, SH-SY5Y. Both cell lines were cultured in Dulbecco’s modified Eagle’s medium (DMEM; Nissui, Tokyo, Japan) supplemented with 10% fetal bovine serum (FBS; Life Technologies, Gaithersburg, MD, USA) at 37°C in 5% CO_2_. Primary mouse cortex neurons (Thermo Fisher Scientific K.K., Tokyo, Japan) were cultured in complete neurobasal medium supplemented with GlutaMax-I and B-27 (Thermo Fisher Scientific K.K.) according to the instruction. Carnosine was purchased from Wako (Osaka, Japan).

### Exosome isolation

ExoQuick Solution (System Biosciences Inc., Mountain View, CA, USA) and MagCapture Exosome Isolation Kit PS (Wako) were used to isolate exosomes from supernatant of Caco-2 cells treated with 1 or 10 mM carnosine, according to the manufacturer’s instructions [10]. The concentration of carnosine to treat Caco-2 cells was determined by referring to the previous reports [11].

### Exosome ELISA

Purification of exosomes was confirmed by PS Capture Exosome ELISA Kit (Wako) according to the manufacturer’s instructions. Antibodies used as capture reagents were as follows: anti-CD63 contained in the above kit, and anti-CD81, anti-CD9 and anti-Hsp70 contained in the EXOAB-KIT-1 (System Biosciences). For detection of the latter three antigens, we used a goat anti-rabbit IgG HRP-conjugated secondary antibody.

### Incorporation of exosome into SH-SY5Y cells

Exosomes (200 ng) were labeled with 2 μM PKH26 using the PKH26 Red Fluorescence Cell Linker Kit (Sigma-Aldrich, St. Louis, MO, USA) for 1 hr at 37°C. After labeling exosomes with PKH26, free PKH26 was removed by ultrafiltration using Vivacon 500 (100,000 MWCO; Sartorius Stedim Biotech GmbH, Goettingen, Germany). Labeled exosomes (200 mg/well) were added to SH-SY5Y cells (2 × 10^4^ cells/well) in a 96-well plate (96 well SensoPlate Plus Microplate, Glass bottom, Greiner Bio-one, Tokyo, Japan), and observed under a confocal microscope (TCS SP8, Leica Microsystems K.K., Tokyo, Japan).

### Quantitative evaluation of neurite growth

After seeding onto a μClear Fluorescence Black Plate (Greiner-Bio One), SH-SY5Y cells were treated with exosomes for 24 h. SH-SY5Y cells were fixed with 4% paraformaldehyde for 15 min, blocked with blocking buffer (1 × PBS, 5% goat serum, 0.3% Triton X-100) for 1 h, and treated with Neuro-Chrom Pan Neuronal Marker (Merck Millipore, Billerica, MA, USA) at 25°C overnight. After washing with PBS, cells were stained with Alexa Fluor 555 F(ab′)_2_ fragment of goat antibody IgG (H+L) (Thermo Fisher Scientific, Waltham, MA, USA) for 1 h. After washing with PBS, cells were further stained with Hoechst 33342 (Dojindo, Kumamoto, Japan) for 15 min, and neurite length was measured using the IN Cell Analyzer 1000 (GE Healthcare, Amersham Place, UK).

### Quantitative reverse transcription polymerase chain reaction

RNA was prepared using the High Pure RNA Isolation kit (Roche Diagnostics GmbH, Mannheim, Germany), and cDNA was prepared using the SuperScript IV Reverse Transcriptase (Thermo Fisher Scientific), as described previously [12]. Quantitative reverse transcription polymerase chain reaction (qRT-PCR) was performed using the Thunderbird SYBR qPCR mix (Toyobo, Osaka, Japan) and Thermal Cycler Dice Real Time System TP-800 (Takara, Shiga, Japan). Samples were analyzed in triplicate, and gene expression levels were normalized to the corresponding glyceraldehyde-3-phosphate dehydrogenase (*GAPDH*) level. PCR primer sequences are shown in S1 Table.

### miRNA microarray assay

Microarray assay of miRNA contained in exosomes derived from non-treated or carnosine-treated Caco-2 cells was performed. Total RNA was isolated from exosomes using TRIzol Reagent (Thermo Fisher Scientific) and purified using miRNeasy Mini Kit (Qiagen, Valencia, CA). Total RNA (100 ng) of each sample was labeled using FlashTag Biotin HSR RNA Labeling Kit (Affymetrix, Santa Clara, CA, USA), and hybridized to a Affimetrix GeneChip miRNA 4.0 Array. Relative hybridization intensities and background hybridization values were calculated using the Affymetrix Expression Console. We processed the raw CEL files for gene-level analysis with median polish summarization and quantile normalization by Affymetrix Transcriptome Analysis Console Software, and obtained normalized intensity values. To identify up- or down-regulated genes, we calculated ratios (non-log scaled fold-change) from the normalized intensities of each gene for comparisons between control and experimental samples. Then, we established criteria for regulated genes: (up-regulated genes) ratio ≥ 1.3-fold, (down-regulated genes) ratio ≤ 0.77 [13]. Target genes for miRNA were predicted using TargetScan (http://www.targetscan.org/vert_71/). To determine significantly over-represented gene ontology (GO) categories and significantly enriched pathways, we used tools and data provided by the Database for Annotation, Visualization, and Integrated Discovery (DAVID, http://david.abcc.ncifcrf.gov) [14,15].

### mRNA microarray assay

Total RNA was isolated from SH-SY5Y cells treated with exosomes derived from non-treated or carnosine-treated Caco-2 cells using Isogen II (Nippon Gene, Tokyo, Japan). The RNA quality was tested as described previously [9]. cRNA was labeled with Low Input Quick Amp Labeling (Agilent Technologies, Santa Clara, CA, USA), and hybridized to DNA microarray (SurePrint G3 Human Gene Expression 8x60K v3, Agilent). Relative hybridization intensities and background hybridization values were calculated using Feature Extraction Software (Agilent).

We identified genes with altered expression, as described previously [9]. We then established criteria for significantly up- or down-regulated genes: up-regulated genes, Z-score ≥ 2.0 and ratio ≥ 1.5-fold; down-regulated genes: Z-score ≤ -2.0 and ratio ≤ 0.66-fold. Significantly over-represented GO categories and enriched pathways were determined as described above. Heat map was generated using MeV software [16], and a hierarchical clustering method was used to sort the genes. Color indicates the distance from the median of each row.

### Pathway analysis

We performed pathway analysis by subjecting selected genes to Ingenuity Pathway Analysis (IPA, Qiagen, Redwood City, CA, USA). Using the Ingenuity Knowledge Base, known functional pathways were tested for enrichment with selected genes. Selected genes were assigned to known functional networks based on canonical pathways, relationship to upstream regulators, molecular and cellular functional grouping, and associated network functions. In IPA, an assignment of nodes in gene network uses published observations stored in the Ingenuity Pathway Knowledge Base.

To obtain additional insight into gene functions, selected genes were examined for their potential role in neuronal cells using NCBI gene (www.ncbi.nlm.nih.gov/gene).

### Expression analysis of miRNA

Exosomes were prepared from supernatant of Caco-2 cells treated with carnosine using the MagCapture Exosome Isolation Kit PS, as described above. Total RNA in the exosomes was isolated using a miRNeasy Serum/Plasma Kit (Qiagen) according to the manufacturer’s protocol. Cellular total RNA was isolated as described above. cDNA against miRNA was synthesized using the miScript II Kit (Qiagen). Primer sequences to amplify miRNA (miR-6769b-5p, 5′-UGGUGGGUGGGGAGGAGAAGUGC-3′; miR-24-3p, 5′-UGGCUCAGUUCAGCAGGAACAG-3′; miR-937-5p, 5′-GUGAGUCAGGGUGGGGCUGG-3′) were designed by Qiagen. qRT-PCR was performed using the miScript SYBR Green PCR Kit (Qiagen) according to the manufacturer’s protocol. The determination of miRNA levels in each sample was performed by the 2^–ΔΔCt^ method [17].

### Transfection of miR mimic and siRNA

The miR-6769b-5p mimic (UGGUGGGUGGGGAGGAGAAGUGC, MSY0027620, Qiagen) and siRNA against ATXN1 (siATXN1_8: CCCTTCTACCCTCAACGACAA, SI04317964, Qiagen; siATXN1_9: AAGCAACGACCTGAAGATCGA, SI04332027, Qiagen) were transfected into SH-SY5Y cells using the HyPerFect Transfection Reagent (Qiagen). Transfection conditions were determined using AllStar Hs Cell Death Control siRNA (Qiagen). After transfection, the expression of miR6769b-5p and *ATXN1* mRNA was determined by qRT-PCR, and the neurite length was quantitatively evaluated as described above.

### Animal experiments

C57/BL6J mice (6 weeks old, male, n = 3) were orally administered carnosine (2 g/L) in drinking water for 7 days. Exosome was prepared from serum by using MagCapture Exosome Isolation Kit PS as described previously. All mouse experiments and protocols were in accordance with the Guide for the Care and Use of Laboratory Animals and were approved by the Ethics Committees on Animal Experimentation (Kyushu University; approved number: A30-244-0).

### Statistical analysis

All experiments were performed at least 3 times, and the corresponding data are shown. The results are presented as mean ± standard deviation. Statistical significance was determined using a two-sided Student’s *t*-test. Statistical significance was defied as P<0.05 (*P<0.05; **P<0.01; ***P<0.001).

## Results

### Isolation of exosomes from the supernatant of carnosine-treated Caco-2 cells

**Fig 1:**
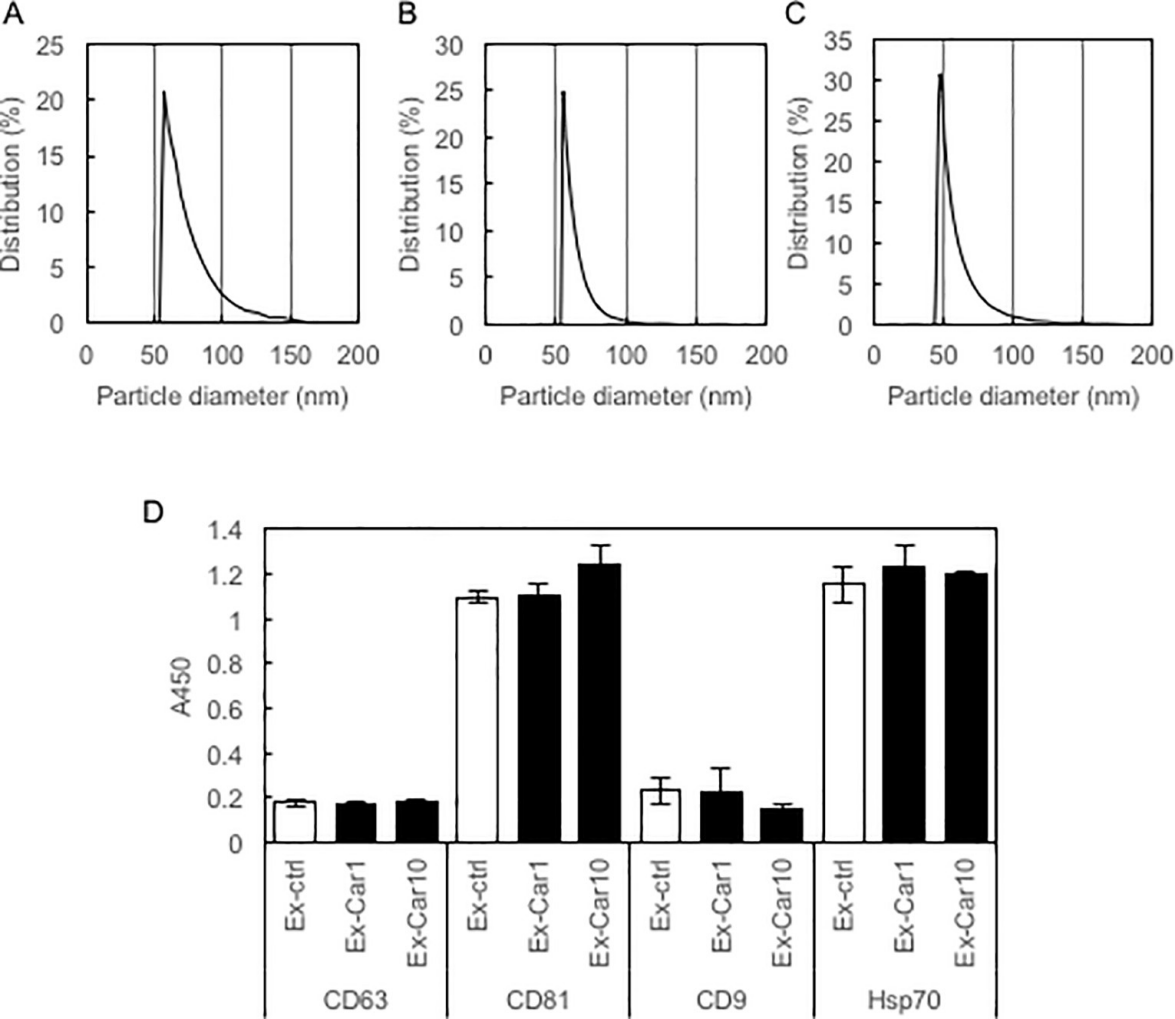
Exosomes isolated from supernatant of Caco-2 cells. Particle sizes of isolated exosomes derived from non-treated Caco-2 cells (A) and those of Caco-2 cells treated with 1 mM carnosine (B) and 10 mM carnosine (C) were determined by dynamic light scattering using ELSZ-0S (Otsuka Electronics). D, Expression of marker proteins of exosome was determined using the PS Capture Exosome ELISA kit (Wako).

Exosomes were isolated from the supernatant of carnosine-treated Caco-2 cells using the MagCapture Exosome Isolation Kit PS. We determined the particle size of isolated exosomes by dynamic light scattering using ELSZ-0S (Otsuka Electronics, Osaka, Japan). Results showed that Caco-2 cells produced exosomes of about 50 nm in diameter, regardless of carnosine treatment (Fig 1A–1C). Furthermore, we tested for the expression of exosome marker proteins using the PS Capture Exosome ELISA Kit. Results showed that all exosomes, regardless of carnosine treatment, expressed exosome marker proteins at almost the same level, although the expression levels of CD63 and CD9 were relatively low (Fig 1D). These results demonstrate that Caco-2 cells produce exosomes of the same size that express marker proteins at almost the same level, regardless of carnosine treatment.

### Incorporation of exosomes into SH-SY5Y cells

In the present study, we speculated that carnosine activates the brain-gut interaction via exosomes produced from carnosine-treated intestinal cells. Firstly, we investigated the incorporation of Caco-2-derived exosomes into SH-SY5Y cells. As shown in S1 Fig, Caco-2-derived exosomes labeled with PKH26 were incorporated into SH-SY5Y cells. No significant difference in the incorporation efficiency of exosomes was observed between Caco-2 cell treatments.

### Activation of SH-SY5Y cells by treatment with Caco-2-derived exosomes

**Fig 2:**
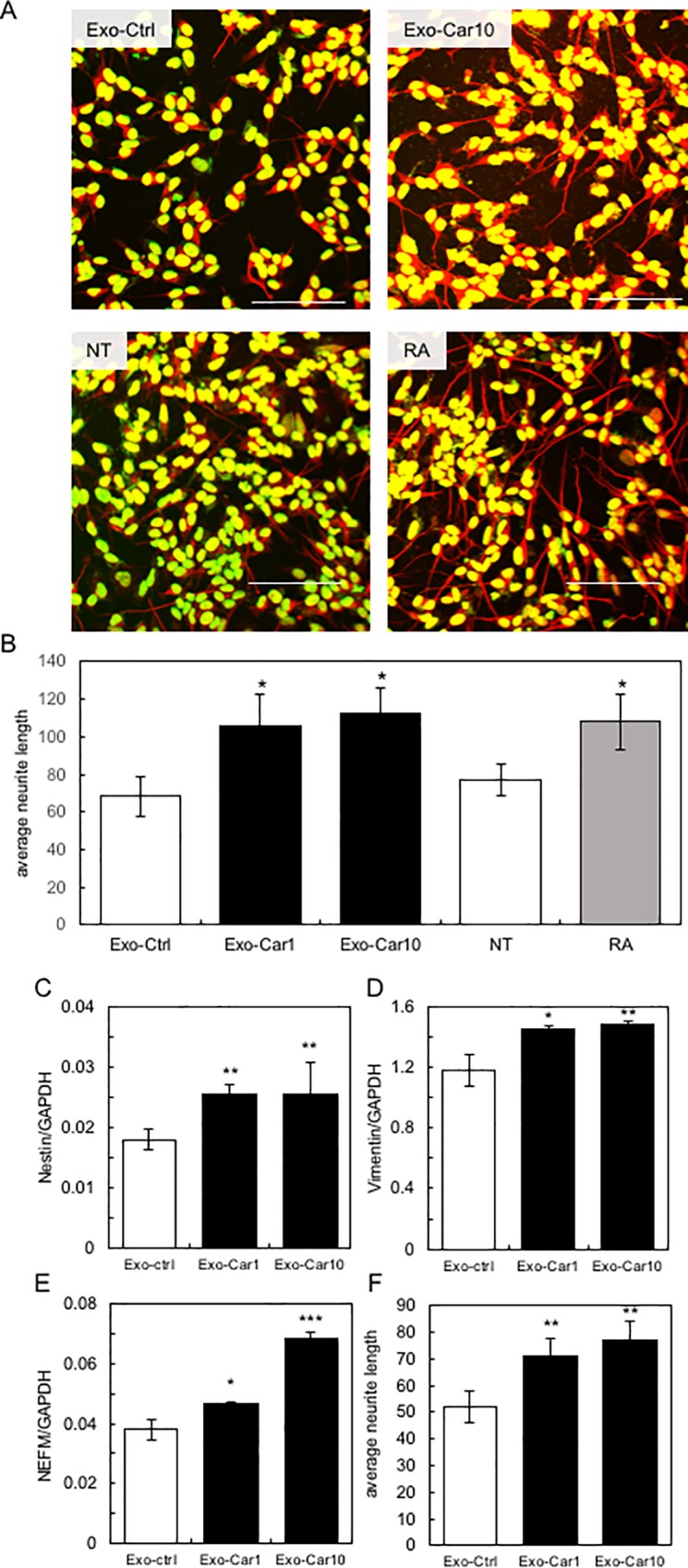
Activation of neuronal cells by Caco-2-derived exosome. A, Neurite growth in SH-SY5Y cells treated with exosomes derived from non-treated Caco-2 cells (Exo-ctrl), from Caco-2 cells treated with 10 mM carnosine (Exo-Car10), and neurite growth in non-treated SH-SY5Y cells (NT) and cells treated with retinoic acid (RA) were shown. B, Neurite growth in SH-SY5Y cells was quantitatively determined using the IN Cell Analyzer 1000. C-E, Expression of neuronal cell marker genes of nestin (C), vimentin (D) and neurofilament medium (NEFM; E) was quantitatively determined by qRT-PCR. Neurite growth in primary mouse cortex neurons treated with exosomes (Exo-ctrl, Exo-Car1 and Exo-Car10) was quantitatively determined using the IN Cell Analyzer 1000 as described above (F).

As shown above, Caco-2-derived exosomes could be incorporated into SH-SY5Y cells. Here, we attempted to evaluate whether Caco-2-derived exosomes induce neurite growth in SH-SY5Y cells. Firstly, we optimized the conditions for neurite growth in SH-SY5Y cells induced by exosomes isolated from the supernatant of carnosine-treated Caco-2 cells. Then, we treated SH-SY5Y cells with exosomes equivalent to 90 ng protein for 1 day, and quantitatively evaluated neurite growth using the IN Cell Analyzer 1000. As shown in Fig 2A, exosomes derived from the supernatant of Caco-2 cells treated with 10 mM carnosine (Exo-Car10) significantly induced neurite growth in SH-SY5Y cells compared with exosomes derived from the supernatant of non-treated Caco-2 cells (Exo-Ctrl). The length and diameter of neurites in SH-SY5Y cells were significantly augmented by treatment with Exo-Car10; this effect was equivalent to that observed when SH-SY5Y cells were treated with retinoic acid (Fig 2A and 2B).

Next, we tested for the expression of neuronal cell marker genes of nestin, vimentin, and neurofilament medium (NEFM). Results showed that Exo-Car1 and Exo-Car10 significantly augmented the expression of these marker genes (Fig 2C–2E). These results indicate that Exo-Car activates and induces neurite growth in SH-SY5Y cells. Furthermore, Exo-Car induced neurite growth in primary mouse cortex neurons as well as neuronal cell line, SH-SY5Y (Fig 2F), suggesting that Exo-Car activates neurons.

### Expression profile analysis of exosome miRNA

**Fig 3:**
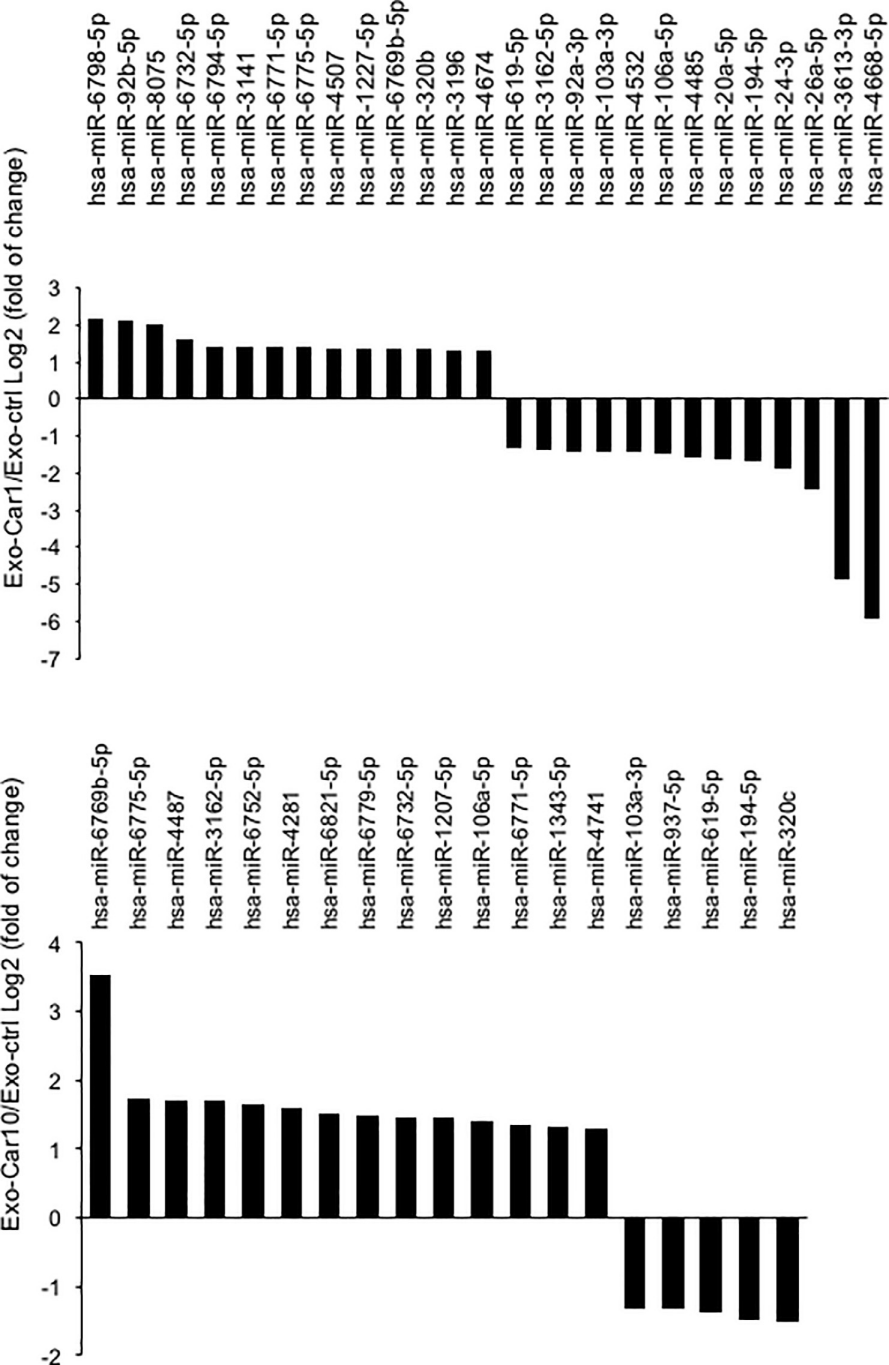
Expression profile analysis of exosome miRNA. After Caco-2 cells were treated with 1 mM or 10 mM carnosine, isolated exosomes were used for miRNA microarray analysis with an Affimetrix GeneChip miRNA 4.0 array. miRNAs with altered expression in response to carnosine treatment are shown.

We next tried to clarify the molecular mechanisms of Exo-Car to activate SH-SY5Y cells, namely miRNA in exosome and its target genes. Firstly, we searched for miRNA with altered expression in Exo-Car and its target genes. After Caco-2 cells were treated with 1 mM or 10 mM carnosine, exosomes were isolated from the supernatant using ExoQuick Solution, as described above. Changes in the expression profile of exosome miRNA in response to carnosine treatment were analyzed using the Affimetrix GeneChip miRNA 4.0 array. The expression of numerous miRNAs was altered in exosomes via treatment with 1 mM or 10 mM carnosine (Fig 3). The expression of 27 miRNAs and 19 miRNAs were altered in Caco-2 cells treated with 1 mM and 10 mM carnosine, respectively. Among these miRNAs, 7 miRNAs (miR-103a-3p, miR-194-5p, miR-619-5p, miR-6732-5p, miR-6769b-5p, miR-6771-5p, and miR-6775-5p) were similarly regulated in Caco-2 cells treated with 1 mM and 10 mM carnosine.

Next, we predicted target genes for these miRNAs using TargetScan, and determined significantly over-represented GO categories and significantly enriched pathways using DAVID. Here, we performed cluster analysis of these target genes to predict the function of miRNA. Results showed that many target genes of the selected miRNA were involved in the development and differentiation of neuronal cells, axon guidance, and neurotrophin signaling pathway, suggesting that these miRNAs contained in the carnosine-treated exosomes activate neuronal cells via their target genes (S2 Table).

### Expression profile analysis of mRNA in the Exo-Car-treated SH-SY5Y cells

Next, in order to identify target genes of Exo-Car in SH-SY5Y cells, we analyzed the expression profile of mRNA in SH-SY5Y cells treated with exosomes. After Caco-2 cells were treated with 1 mM and 10 mM carnosine, exosomes (Exo-Car1 and Exo-Car10) were isolated as described previously. Total RNA was prepared from SH-SY5Y cells treated with these exosomes and with RA as a positive control, and used for DNA microarray analysis. Compared with SH-SY5Y cells treated with exosomes isolated from non-treated Caco-2 cells (Exo-Ctrl), 585 genes and 579 genes showed altered expression in SH-SY5Y cells treated with Exo-Car1 and Exo-Car10, respectively. Furthermore, as shown in the heat map (S2 Fig), many genes showed similar expression profiles between SH-SY5Y cells treated with Exo-Car1, Exo-Car10, and RA. We speculate that genes with altered expressions in Exo-Car-treated SH-SY5Y cells are under the control of miRNA with altered expression in Exo-Car, and involved in the activation of SH-SY5Y cells.

### Integrated analysis

We then performed integrated analysis of miRNAs with altered expression in exosomes in response to carnosine treatment and of mRNAs with altered expression in SH-SY5Y cells in response to Exo-Car treatment. Further, we attempted to identify miRNAs and their target genes involved in the activation of SH-SY5Y cells.

Firstly, we compared target genes of miRNA with altered expression in Exo-Car to mRNAs with altered expression in Exo-Car-treated SH-SY5Y, and identified 137 and 77 typical genes for 1 mM and 10 mM carnosine treatment, respectively. We then selected 14 genes that have been reported to activate and/or maintain neuronal cells using the NCBI database (https://www.ncbi.nlm.nih.gov/pubmed/) (Table 1). Some of other genes were related to the activation of immune cells and maintenance of adipose tissue. Next, we analyzed the interaction network of these genes by in silico analysis (Ingenuity Pathway Analysis (IPA)) to select possible genes of significance and their regulating miRNAs (Table 2, S3 Fig).

**Table 1:** Neuronal gene and its regulating miRNA selected by integration analysis.

Gene name	Exo-Car1 (Z score)	Exo-Car10 (Z score)	Regulatory miRNA	Function
ATXN1	-16.404	-15.972	miR-6769b-5p	neurodegenerative disorders
			miR-6794-5p	
SLITRK5	-13.012	-12.765	miR-6769b-5p	neurite-modulating activity
			miR-3196	
			miR-4507	
ATCAY	1.609	–	miR-24-3p	neuron-restricted protein
			miR-619-5p	
			miR-937-5p	
SNCAIP	6.362	–	miR-24-3p	interacts with α-synuclein in neuronal tissue
CPLX2	5.243	–	miR-194-5p	function in synaptic vesicle exocytosis
			miR-619-5p	
NRSN1	2.599	–	miR-4668	transduction of nerve signals
KIRREL3	-8.776	–	miR-6769b-5p	expressed in adult brain
KCNG4	12.3	–	miR-24-3p	regulating neurotransmitter release
			miR-4668	
			miR-619-5p	
NOTCH1	1.66	–	miR-24-3p	receptor for neuronal DNER
NRXN1	2.062	–	miR-3162	the neurexin family
PHOX2A	1.857	–	miR-3162	role in development of nervous system
AVP	-1.62	–	miR-3196	transported axonally to the nerve endings
LHX3	1.626	–	miR-4532	development and motor neuron specification
VAMP4	–	1.663	miR-103a-3p	support distinct forms of neurotransmission

**Table 2:** Selected miRNA and target gene.

miRNA	Target gene
miR-6769b-5p	ATXN1, KIRREL3, SLITRK5
miR-619-5p	ATCAY, CPLX2, KCNG4
miR-24-3p	ATCAY, KCNG4, NOTCH1, SNCAIP
miR-937-5p	ATCAY

### Expression profile of miRNA

**Fig 4:**
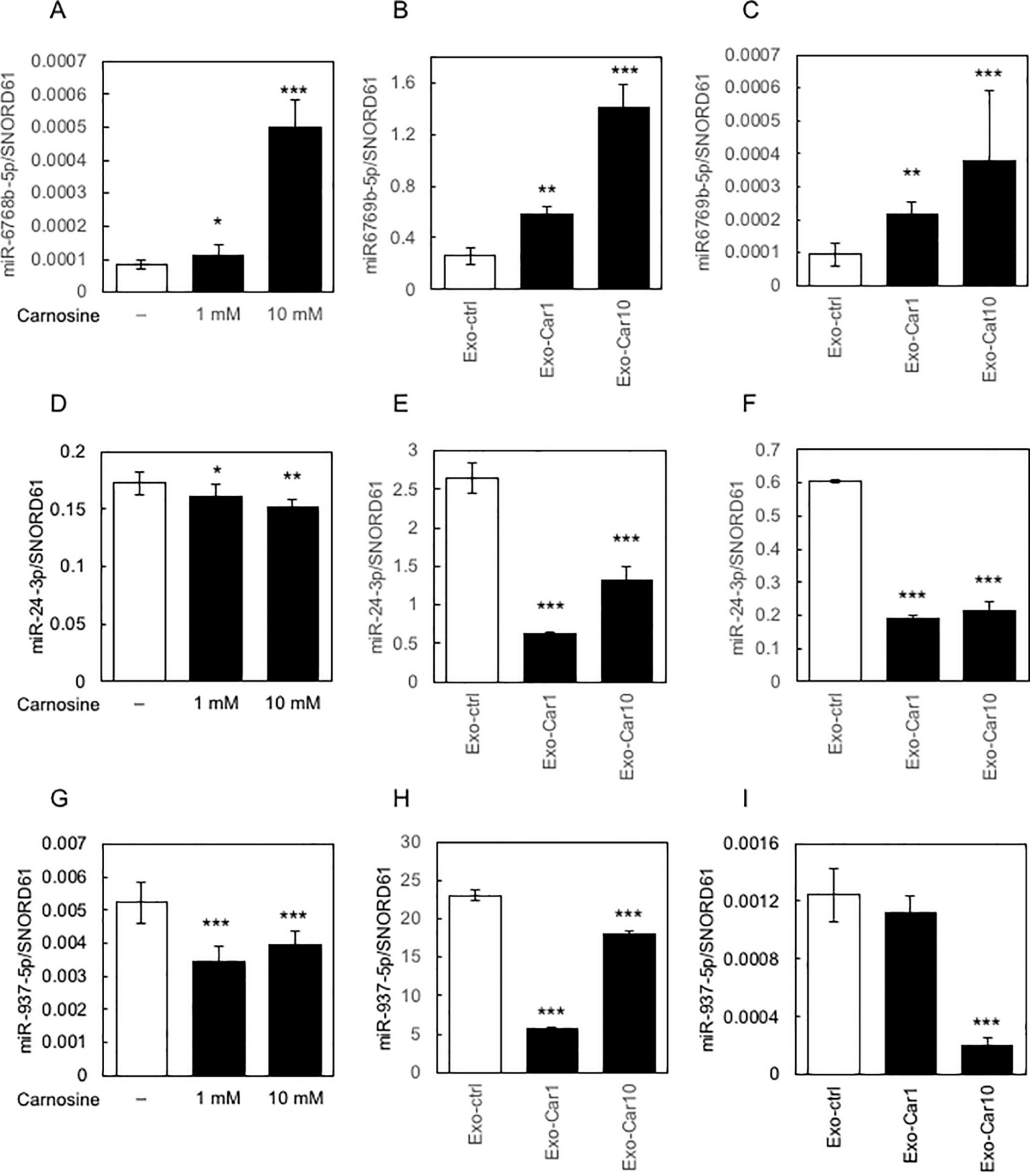
Expression profile of miRNA. The changes in the expression of the selected miRNA (miR-6769b-5p, miR-24-3p, and miR-937-5p) in Caco-2 cells treated with carnosine are shown in A, D, and G, respectively. The changes in the expression of miRNA in cells treated with Exo-Car are shown in B (miR-6769b-5p), E (miR-24-3p) and H (miR-937-5p). The changes in the expression of miRNA (C, miR-6769b-5p; F, miR-24-3p; I, miR-937-5p) in SH-SY5Y cells treated with Exo-Car are shown.

Firstly, we tested for changes in expression of the selected miRNA (miR-6769b-5p, miR-24-3p, miR-937-5p, and miR-619-5p) in Caco-2 cells treated with carnosine. The expression of miR-6769b-5p was up-regulated, while that of miR-24-3p and miR-937-5p was down-regulated in Caco-2 cell treated with carnosine; however, that of miR-619-5p remained unchanged (Fig 4A, 4D and 4G).

Next, we tested for changes in expression of these miRNAs in Exo-Car-treated cells. After preparing the Exo-Car, we quantitatively determined the expression level of miRNAs. Consistent with the results above (Fig 4A, 4D and 4G), the expression of miR-6769b-5p was up-regulated, and that of miR-24-3p and miR-937-5p was down-regulated in Exo-Car (Fig 4B, 4E and 4H). The change in the expression of miR-619-5p in Exo-Car was not significant (S4 Fig).

Finally, we tested for changes in the expression of these miRNAs in SH-SY5Y cells treated with Exo-Car. After preparing Exo-Car from the supernatant of carnosine-treated Caco-2 cells, SH-SY5Y cells were treated with the Exo-Car and changes in the expression of these miRNA in SH-SY5Y cells were quantitatively determined by qRT-PCR. Results demonstrated that the expression of miR-6769b-5p was up-regulated, and that of miR-24-3p and miR-937-5p was down-regulated in SH-SY5Y cells treated with Exo-Car (Fig 4C, 4F and 4I). The expression of miR-619-5p remained unchanged in SH-SY5Y cells even after treatment with Exo-Car.

### Expression profile of target genes in Exo-Car-treated SH-SY5Y cells

**Fig 5:**
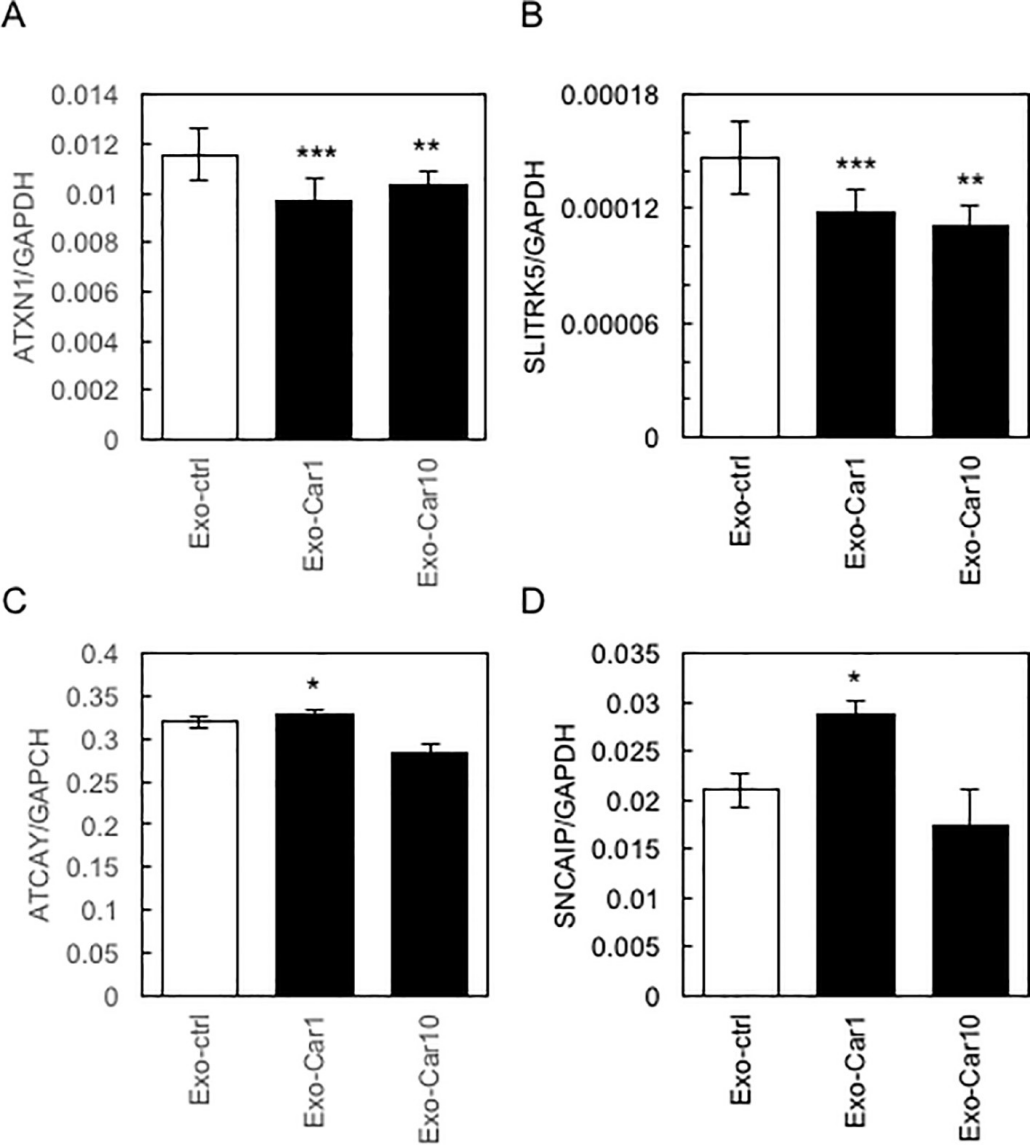
Expression profile of target genes in Exo-Car-treated SH-SY5Y cells. The expression profile of target genes of miRNA in Exo-Car-treated SH-SY5Y cells, as determined by qRT-PCR, is shown (A, ATXN1; B, SLITRK5; C, ATCAY; D, SNCAIP).

Next, we selected several genes among target genes of miRNA (Tables 1 & 2) and analyzed the expression profiles of these genes in Exo-Car-treated SH-SY5Y cells (Fig 5). Among these target genes, *ATXN1* and *SLITRK5* were significantly down-regulated in Exo-Car-treated SH-SY5Y cells (Fig 5A and 5B). The expression of *ATCAY* and *SNCAIP* was up-regulated only in Exo-Car1-treated cells (Fig 5C and 5D). Thus, we focused on miR-6769b-5p, which regulates the expression of *ATXN1* and *SLITRK5*.

### The involvement of miR-6769b-6-5p and ATXN1 in neurite growth in SH-SY5Y cells

**Fig 6:**
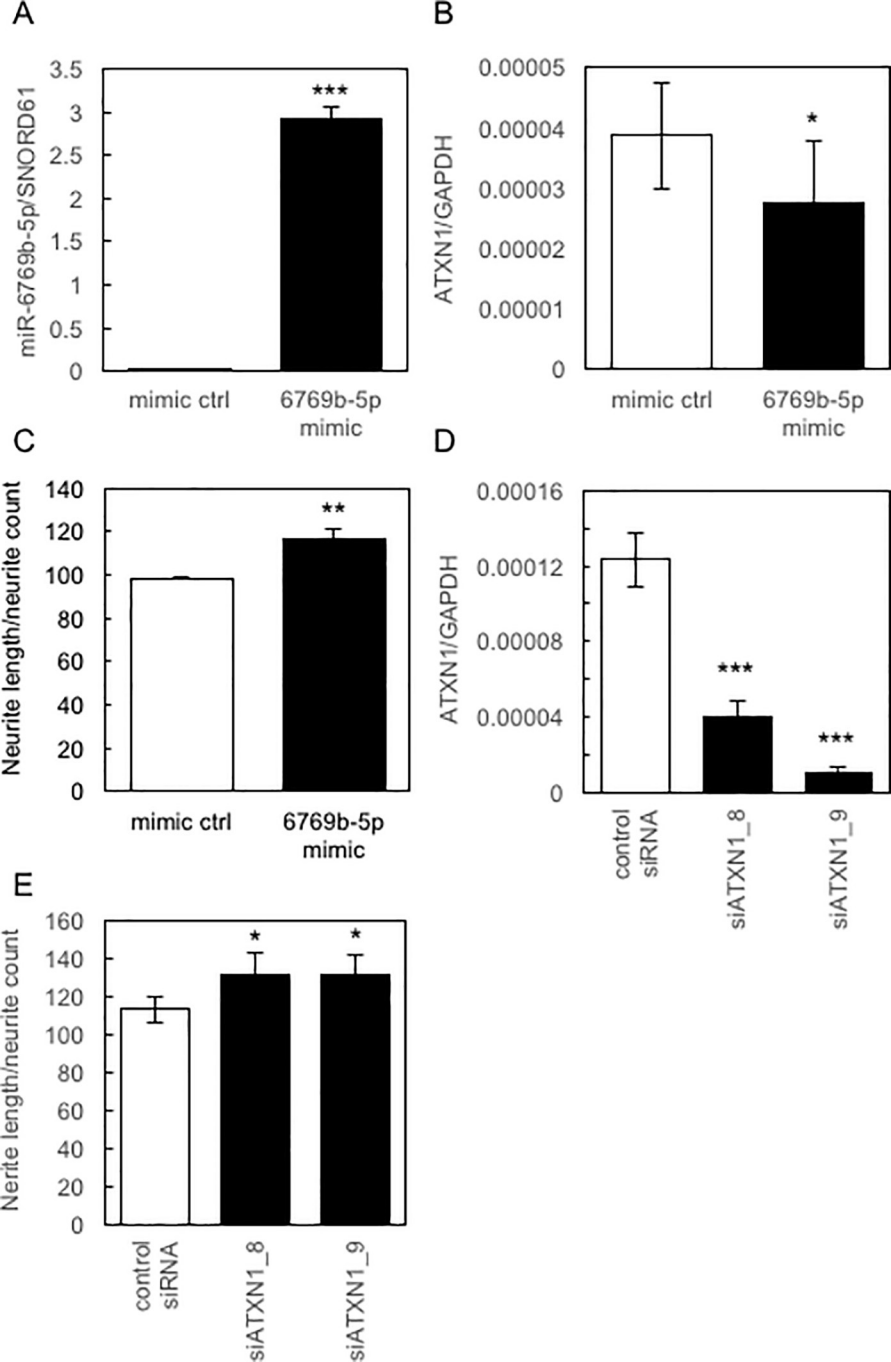
Involvement of miR-6769b-5p and *ATXN1* in neurite growth in SH-SY5Y cells. The expression of miR-6769b-5p (A) and *ATXN1* (B), and neurite growth (C) in SH-SY5Y cells transduced with miR-6769b-5p mimic was determined by qRT-PCR and by using the IN Cell Analyzer 1000, respectively. D, The expression of *ATXN1* in SH-SY5Y cells transduced with siRNA targeting ATXN1 (siATXN1_8 and siATXN1_9) were determined by qRT-PCR. E, Neurite growth in SH-SY5Y cells transduced with siATXN1_8 and siATXN1_9 was determined using the IN Cell Analyzer 1000.

To evaluate the role of miR-6769b-5p in the regulation of *ATXN1* expression and neurite growth in SH-SY5Y cells, we used miRNA mimics with similar functions to miR-6769b-5p. As shown in Fig 6A–6C, the miR-6769b-5p mimic was efficiently transduced into the SH-SY5Y cells, resulting in the significant down-regulation of the expression of *ATXN1* and induction of neurite growth in SH-SY5Y cells. These results suggest that the decreased expression of *ATXN1* induces neurite growth in SH-SY5Y cells. To confirm this, we used siRNA targeting *ATXN1* (siATXN1_8 and siATXN1-_9). Results clearly showed that siATXN1s efficiently down-regulated the expression of *ATXN1* in SH-SY5Y cells (Fig 6D), which significantly induced neurite growth in SH-SY5Y cells (Fig 6E). These results suggest that decreased expression of *ATXN1* induced by miR-6769b-5p contained in Exo-Car triggered neurite growth in SH-SY5Y cells.

**Fig 7:**
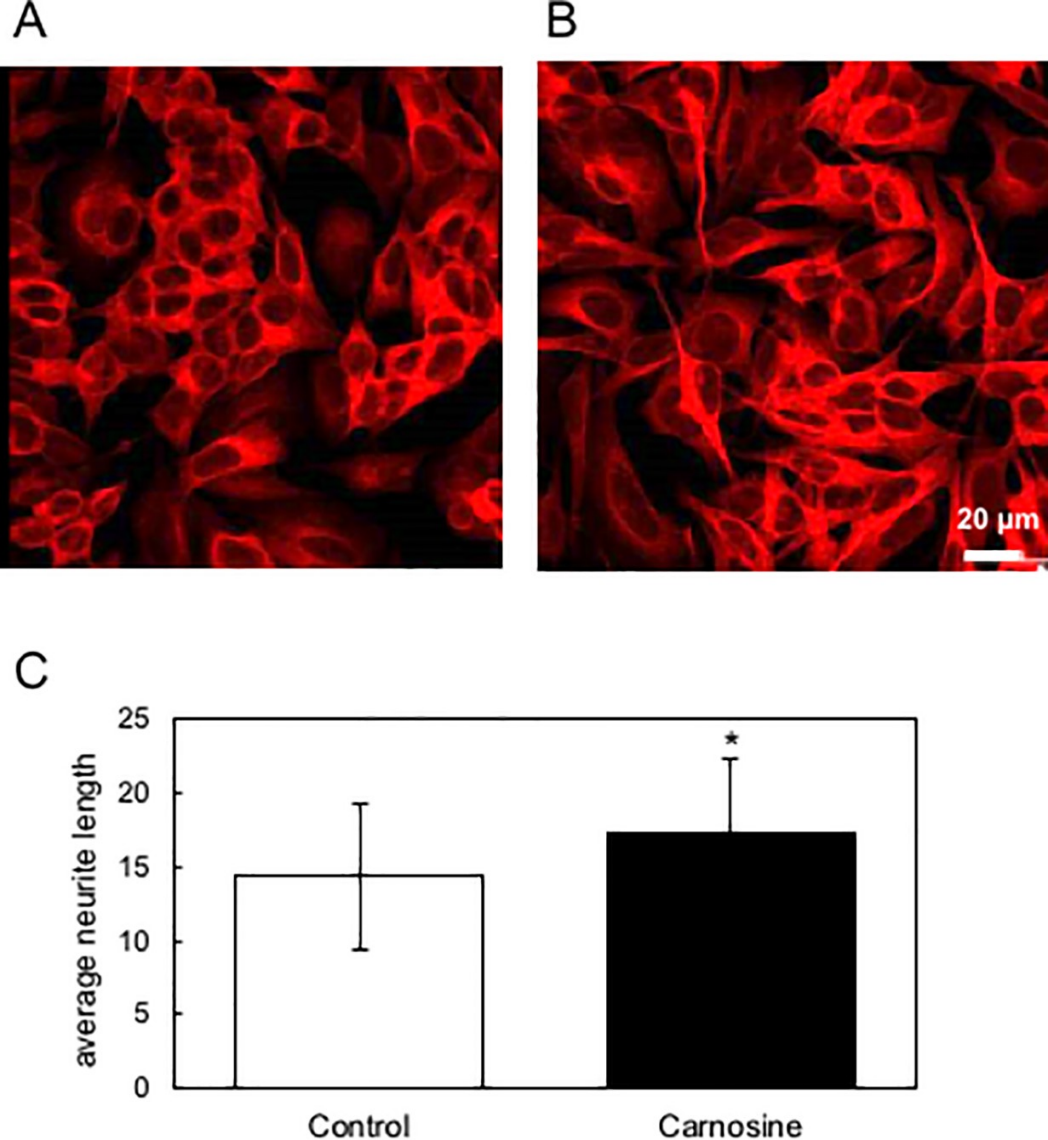
Serum exosome of mice orally administered with carnosine activates neuronal cells. Exosome was prepared from mice orally administered drinking water containing carnosine (B) or not (A) by using MagCapture Exosome Isolation Kit PS as decribed previously. Neurite growth in SH-SY5Y cells treated with these exosomes was quantitatively determined using the IN Cell Analyzer 1000.

Finally, we tested whether serum exosome in mice orally administered carnosine can activate neuronal cells. As shown in Fig 7, serum exosome derived from mice orally administered carnosine significantly extended neurite in SH-SY5Y cells, suggesting that carnosine intake induces exosome that activates neuronal cells in serum. We would like to clarify the molecular mechanisms for generation and functionalities of the carnosine-induced serum exosome in the future study.

## Discussion

Numerous food components have been reported to possess the ability to affect cognitive function and emotion; these include omega-3 fatty acids, curcumin, flavonoids, and resveratrol [18]. Several gut hormones and regulators of synaptic plasticity, such as brain-derived neurotrophic factor, have been reported to affect brain function. In this study, we presented a novel mechanism of action of such a “brain food”, namely carnosine. Until now, we showed that carnosine induces the secretion of several cytokines including BDNF, which would result in the activation of brain-gut interaction and its consequent brain function [9]. Here, we demonstrated that carnosine induced the secretion of exosomes from intestinal epithelial cells, and that the resulting exosomes and its miRNA activated neuronal cells, resulting in the activation of brain function.

Exosomes have received much interest since their key roles in intercellular/intertissue communication has been emerged. Numerous studies have focused on the exosome as a transporter of distinct bioactive molecules such as miRNA, mRNA, and protein, as well as on its therapeutic applications. In particular, exosomes derived from food are of interest owing to their ability to modulate cellular functions. Exosomes are known to be contained in numerous foods including fruits, milk, nuts, seeds, and roots [19]. However, there are a limited number of reports describing the functionality of exosomes whose secretion is induced by food factors. Curcumin has been reported to ameliorate endolysosomal cholesterol accumulation by shuttling cholesterol out of the cells via exosome secretion [20]. However, the involvement of exosomes in the food-induced activation of brain-gut interactions, which is speculated to represent an important molecular mechanism underlying the effects of diet on brain function, has not been reported to date.

In the present study, we sought to elucidate the molecular basis of exosome-mediated activation of neuronal cells by integrated analysis of target genes of differentially expressed miRNAs in exosomes derived from carnosine-treated Caco-2 cells and of differentially expressed mRNAs in cells treated with these exosomes. This integrated analysis, subsequent database screening for genes that activate and maintain neuronal cells, and pathway analysis using IPA enabled the successful identification of target genes and their regulatory miRNAs. Enerly et al. described roles for miRNAs in primary breast tumors by miRNA-mRNA integrated analysis in a previous study [21]; however, to our knowledge, the present study is the first to identify both miRNAs in exosomes and target mRNAs in recipient cells of exosomes via integrated analyses.

To date, studies have revealed that the miR-17-92 cluster and miR-124 function to elongate neurites [22,23]. Here, we identified a novel miRNA, miR-6769-5p, that activates and elongates neurites in SH-SY5Y cells. Furthermore, we identified *ATXN1* as one of target genes of miR-6769-5p. *ATXN1* has been reported to be involved in the pathophysiology of cerebellar seizures [24,25], and further that the silencing of *ATXN1* mRNA provides therapeutic benefits in a mouse model of polyglutamine expansion diseases [26]. In this study, we found that miR-6769-5p-mediated reduction of *ATXN1* expression led to the extension of neurites in neuronal cells and possibly to the activation of brain-gut interaction mediated by carnosine-induced exosomes. MiR-6967-5p and *ATXN1* may therefore represent key factors involved in the activation of brain-gut interaction mediated by specific food components. Next, we need to clarify whether other brain foods activate brain-gut interaction via miR-6769-5p and ATXN1. Furthermore, it would become evident in the future study that various intercellular/intertissue interactions elicited by food components are mediated by functional exosomes.

## Supporting information

S1 Fig Incorporation of exosomes into SH-SY5Y cells. The incorporation of exosomes labeled with PKH26 (red arrow) was observed under a confocal microscope (TCS SP8, Leica Microsystems).
(TIFF)

S2 Fig Expression profile analysis of mRNA in SH-SY5Y cells treated with Exo-Car. Total RNA from SH-SY5Y cells treated with exosomes derived from non-treated Caco-2 cells or Caco-2 cells treated with 1 mM or 10 mM carnosine were used for DNA microarray analysis with the SurePrint G3 Human Gene Expression 8x60K v3 array. The heat map was shown.
(TIFF)

S3 Fig Ingenuity pathway analysis. Representative interaction network of target genes of miRNA with altered expression in Exo-Car and mRNAs with altered expression in Exo-Car-treated SH-SY5Y revealed by in silico analysis.
(TIFF)

S4 Fig Expression profile of miRNA. The changes in the expression of miR-619-5p in Caco-2 cells treated with carnosine (A). The changes in the expression of miR-619-5p in cells treated with Exo-Car are shown in B. The changes in the expression of miR-619-5p in SH-SY5Y cells treated with Exo-Car are shown in C.
(TIFF)

S1 Table Sequence primers. (PDF)

S2 Table Functional annotation cluster of target genes of miRNA with altered expression in response to carnosine treatment. (PDF)
