# Supplementary figures and images for: Carnosine induces intestinal cells to secrete exosomes that activate neuronal cells
Source: PLoS One. 2019 May 28;14(5):e0217394. doi: 10.1371/journal.pone.0217394 (PMC6538158; doi:10.1371/journal.pone.0217394)

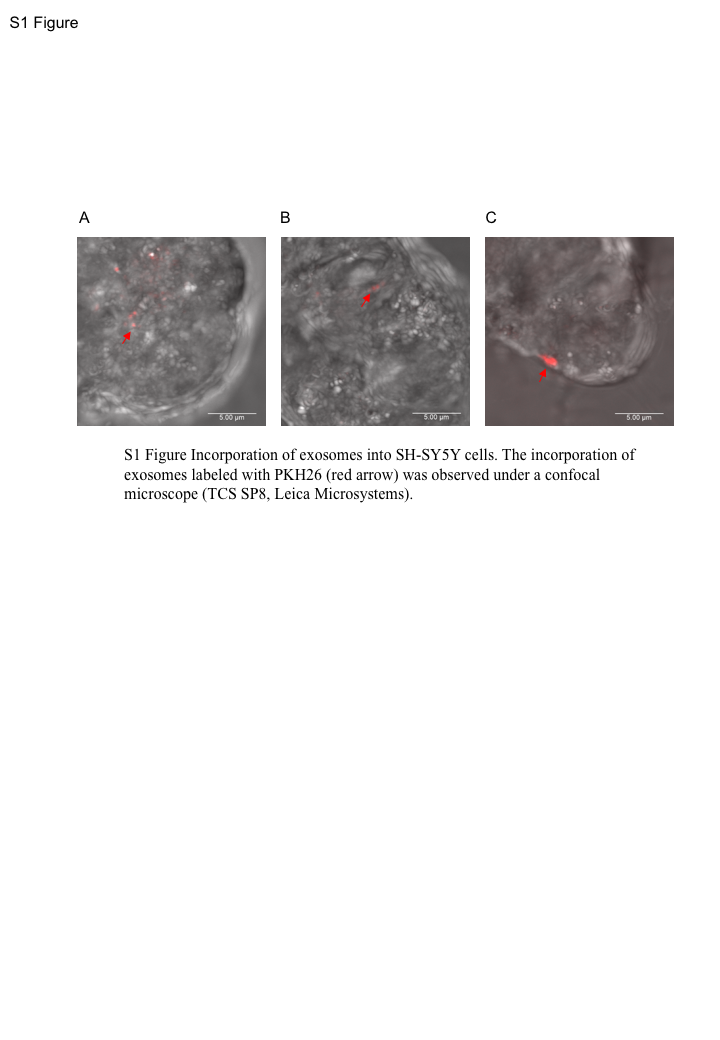

Supplement: S1 Fig — The incorporation of exosomes labeled with PKH26 (red arrow) was observed under a confocal microscope (TCS SP8, Leica Microsystems). (TIFF) [file pone.0217394.s001.tiff]

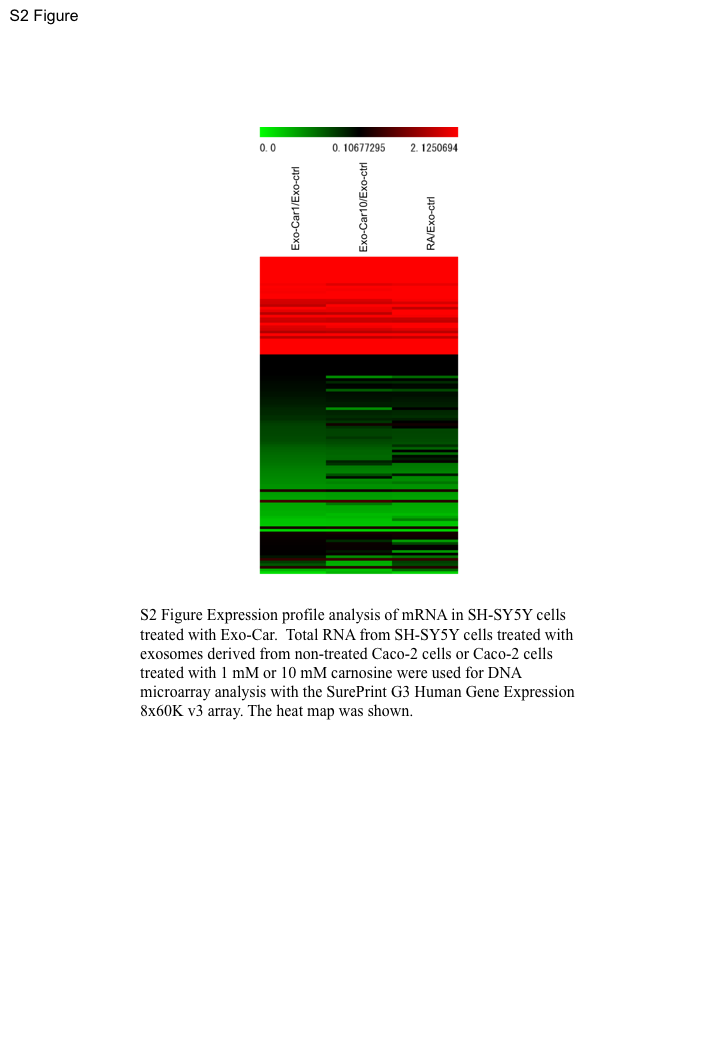

Supplement: S2 Fig — Total RNA from SH-SY5Y cells treated with exosomes derived from non-treated Caco-2 cells or Caco-2 cells treated with 1 mM or 10 mM carnosine were used for DNA microarray analysis with the SurePrint G3 Human Gene Expression 8x60K v3 array. The heat map was shown. (TIFF) [file pone.0217394.s002.tiff]

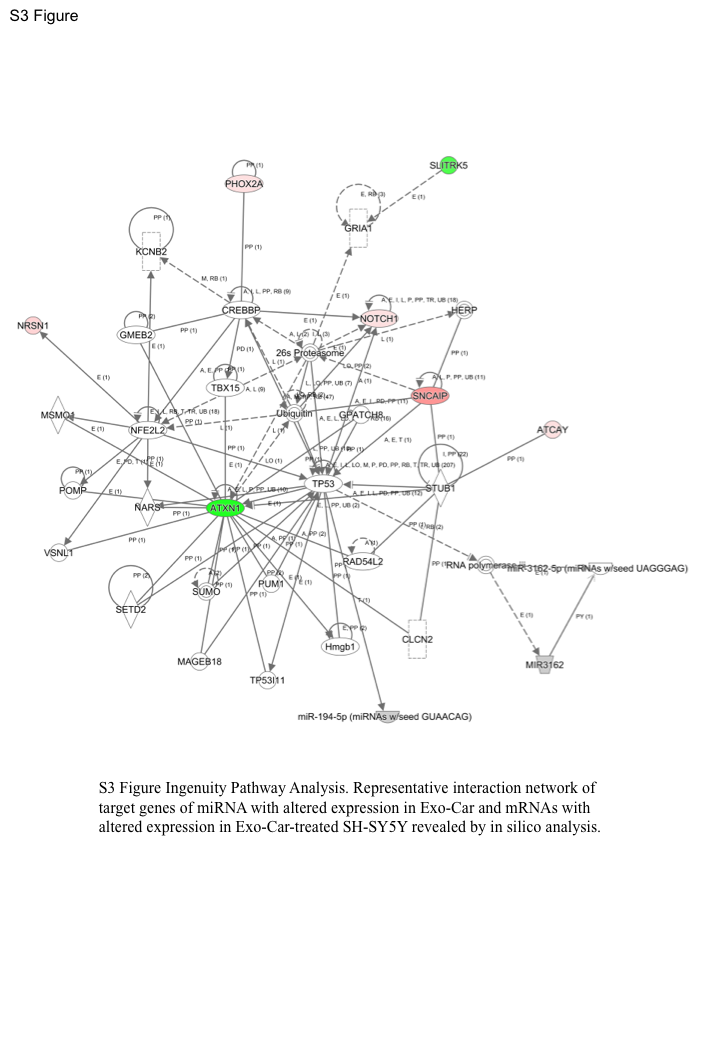

Supplement: S3 Fig — Representative interaction network of target genes of miRNA with altered expression in Exo-Car and mRNAs with altered expression in Exo-Car-treated SH-SY5Y revealed by in silico analysis. (TIFF) [file pone.0217394.s003.tiff]

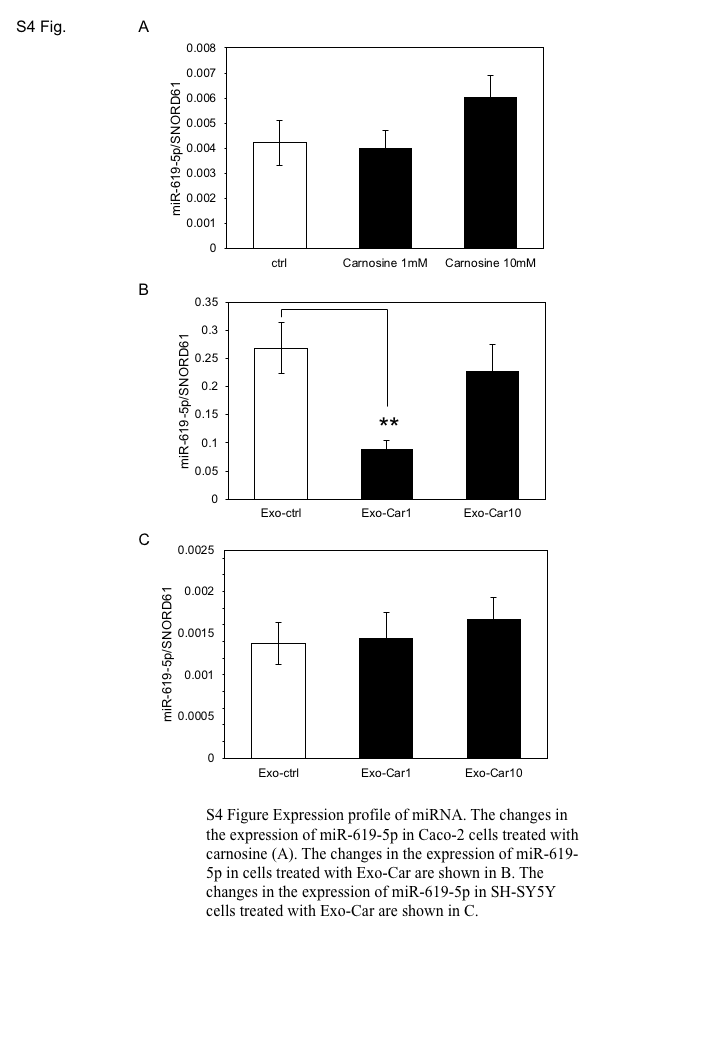

Supplement: S4 Fig — The changes in the expression of miR-619-5p in Caco-2 cells treated with carnosine (A). The changes in the expression of miR-619-5p in cells treated with Exo-Car are shown in B. The changes in the expression of miR-619-5p in SH-SY5Y cells treated with Exo-Car are shown in C. (TIFF) [file pone.0217394.s004.tiff]
