# Supplementary material for: Carnosine induces intestinal cells to secrete exosomes that activate neuronal cells
Source: PLoS One. 2019 May 28;14(5):e0217394. doi: 10.1371/journal.pone.0217394 (PMC6538158; doi:10.1371/journal.pone.0217394)
Supplement: S1 Table — (PDF) [file pone.0217394.s005.pdf]

S1 Table Sequence primers

Target	Sequence
GAPDH mRNA	5'-CGTGGAAGGACTCATGAC-3'
	5'-CAATTCGTTGTCATACCAG-3'
Nestin mRNA	5'-GAGAGGGAGGACAAAGTCCC-3'
	5'-TCCCTCAGAGACTAGCGCAT-3'
Vimentin mRNA	5'-GTTTCCAAGCCTGACCTCAC-3'
	5'-GCTTCAACGGCAAAGTTCTC-3'
NEFM mRNA	5'-AGACATCCACCGGCTCAAGG-3'
	5'-CGACGCCTCCTCGATGTCTT-3'
ATXN1 mRNA	5'-AGATATGGGTGAGGAAGCAGAGC-3'
	5'-CAGTCTGTTGAGCTGCTTGTGG-3'
SLITRK5 mRNA	5'-TCGGTGCCCTTGTCTGTGTT-3'
	5'-TCGGAGTTGTTGGTGCTGGT-3'
ATCAY mRNA	5'-GTCAGCAGCCCATCCTTCCA-3'
	5'-CGACGTCTTAACATCACGACCG-3'
SNCAIP mRNA	5'-ACAACACCAGACTGCCAGCT-3'
	5'-TTCTTCTGGGCCTTGGGAGC-3'
