# Supplementary material for: Carnosine induces intestinal cells to secrete exosomes that activate neuronal cells
Source: PLoS One. 2019 May 28;14(5):e0217394. doi: 10.1371/journal.pone.0217394 (PMC6538158; doi:10.1371/journal.pone.0217394)
Supplement: S2 Table — (PDF) [file pone.0217394.s006.pdf]

S2 Table Functional annotation cluster of target genes of miRNA with altered expression in response to carnosine treatment

miRNA	Functional annotation cluster of target gene	miRNA	Functional annotation cluster of target gene
has-miR-24-3p	neurotrophin TRK receptor signaling pathway	has-miR-4281	neurotrophin signaling pathway
	axon guidance		axon guidance
has-miR-26-5p	postsynaptic density	has-miR-4487	neural tube development
	neuron differentiation		neurotrophin signaling pathway
	neuron projection development	has-miR-4507	regulation of neuron differentiation
	axon guidance		neurotrophin signaling pathway
	neurotrophin signaling pathway		axon guidance
has-miR-92b-5p	neuron differentiation	has-miR-4532	neuron differentiation
	neuron development		neuron development
	neuron projection morphogenesis		postsynaptic density
	axon guidance		axonogenesis
	neurotrophin signaling pathway	has-miR-4668-5p	neural tube foration
has-miR-103a-3p	neuron development		astrocyte development
	neuron projection		regulation of neurogenesis
	axonogenesis		regulation of neuron differentiation
	axon cargo transport	has-miR-4674	neurotrophin signaling pathway
has-miR-194-5p	neurogenesis	has-miR-4741	axon guidance
	axon guidance		neurotrophin signaling pathway
	neural tube development	has-miR-6769b-5p	neural tube development
	neuron projection		axon guidance
has-miR-320b	neuron projection		regulation of nervous system development
	neurotrophin signaling pathway		regulation of neuron differentiation
	neuron projection morphogenesis		neurotrophin signaling pathway
	axon guidance	has-miR-6732-5p	neuron projection
has-miR-937-5p	axonogenesis		primary neural tube formation
	neuron projection morphogenesis		neuron differentiation
	neuron development		neuron projection morphogenesis
has-miR-1207-5p	neural tube development		neurotransmitter transport
	neurotrophin signaling pathway		axon guidance
has-miR-1227-5p	regulation of neuron projection development		neurotrophin signaling pathway
	regulation of axonogenesis	has-miR-6771-5p	axonogenesis
	regulation of nervous system development	has-miR-6775-5p	neuron differentiation
	neurotrophin signaling pathway		neuron projection morphogenesis
has-miR-1343-5p	neurotrophin signaling pathway		axonogenesis
has-miR-3141	central nervous system neuron differenatioin		regulation of neurotransmitter levels
	regulation of axonogenesis	has-miR-6794-5p	axon guidance
	Alzheimer's disease		neurotrophin signaling pathway
has-miR-3162-5p	postsynaptic density	has-miR-6798-5p	axon guidance
	neuron projection development	has-miR-6821-5p	regulation of neuron differentiation
	neuron differentiation		regulation of nervous system development
	neuron projection morphogenesis		neurotransmitter transport
	axonogenesis		neuron apoptosis
	synaptic transmission	has-miR-8075	primary neural tube formation
	neurotrophin signaling pathway		
